# Editorial: Advancements and challenges in developmental psychopathology and mental health

**DOI:** 10.3389/frcha.2023.1271736

**Published:** 2023-09-14

**Authors:** Maria Melchior

**Affiliations:** ^1^ Department of Social Epidemiology, Institut National de la Santé et de la Recherche Médicale (INSERM), Paris, France; ^2^ Faculté de Médecine, Sorbonne Universités, Paris, France

**Keywords:** developmental psychopathology, mental health, children, adolescents, etiology, measurement

**Editorial on the Research Topic** Advancements and challenges in developmental psychopathology and mental health

Child and adolescent development and mental health is a broad and multidisciplinary area of research and medical as well as medical and psychosocial care. The articles published in the Editor's Showcase exemplify current trends in the field and suggest directions for new research.

First, in the 21st century there still are debates about how to define and measure psychiatric disorders among children and adolescents. One particular issue is identifying as precociously as possible early psychosis which has devasting effects on children's long-term health and social trajectories, yet remains exceedingly difficult to predict and treat early (1). Hamasaki et al. show evidence that the Child Psychosis-risk Screening System is valid to identify children and adolescents who have early psychotic symptoms and distinguish them from those who experience neurodevelopmental problems. This is a fundamental first step to better understand this outcome. The next step is to identify the prevalence and associated risk factors to target research as well as healthcare efforts. On an entirely different mental health topic, Lesinskienė et al. conducted a study evaluating the frequency of pica, an eating disorder in which individuals feel an urge to eat non nutritious and non-food items. In an online study of 594 persons, the parents of 3.7% of children reported that their child ate non-food items between ages 3 and 5 years, this tendency being associated with sensory sensitivity, a trait identified as predicting later emotional difficulties (2). Thus, investigating children's sensitivity to their environment may be a way of identifying risk of psychological distress as well as rare eating disorders such as pica. While studies published in this Research Topic mostly rely upon information provided by parents and youths, the paper authored by Peck et al. and based in Australia shows that routinely collected data to identify children at risk of mental health problems. Combining police and health records, the authors demonstrate that among a sample of 775 adolescents charged with acts of violence against their parents, a majority had a previous contact with the mental health care system, mostly due to symptoms of anxiety and stress. Additionally, many had been exposed to family violence and substance use. These results shed light upon the richness of data collected via multiple services that can be in contact with young people and their families, that can serve to identify those at high risk.

Second, child and adolescent psychopathology risk at the individual and at the population level is influenced by a variety of risk factors and exposures, ranging from genes to political upheaval. In a study of 630 twins born in Québec, Canada between 1995 and 1998 and followed-up over time, Plamondon et al. show that symptoms of inattention in preschool are related to children's later academic achievement, and that part of this association appears to due to genetic factors. However environmental factors—shared and especially non-shared, play a more significant role. Moreover, genetic factors do not seem to explain the shared variance between symptoms of inattention and later depression, pointing once more to the role of early life experiences and exposures in wiring individuals' long-term functioning. While genes are likely to play a role in the occurrence and persistent of the most severe and comorbid forms of attention deficit and hyperactivity disorder (3), additional research using precise measures of children's environments—in the family but also at school and in their neighborhood at different points in time is needed to better document and understand the role of non-genetic risk factors (4). The family is generally thought to play a less important role in adolescence than in childhood, yet Wright et al. show that parental social support remains key when youths experience symptoms of depression. This appears to be especially the case among girls, possibly indicating sex-specific ways in which depression is expressed. Finally, on the opposite end of the etiological spectrum from genetics, Sun et al. contribute to the scientific literature showing the deleterious impact of the COVID-19 pandemic and preventive measures which were taken to control the spread of the SARS-Cov-2 virus, such as lockdowns and school closures on children (5). Studying primary school-aged children in the UK, they report that those who had been born very preterm, were at increased risk of experiencing emotional distress during the COVID-19 pandemic, probably in part because of higher levels of pre-existing internalizing symptoms. These results are consistent with research conducted in other settings during the COVID-19 pandemic which showed that children born very preterm (6), as well as those who had parents who experienced mental health difficulties themselves or had financial difficulties, were most likely to experience psychological difficulties in the context created by the pandemic and resulting sanitary crisis (7, 8).

Overall, the articles published in this Research Topic demonstrate the breadth and the richness of research in child and adolescent mental health around the world, the contributions being truly global. Let these first publications give the readers a rapid overview of all the topics that can be addressed in the Section of Developmental Psychopathology and Mental Health of Frontiers in Child and Adolescent Psychiatry, using a variety of study designs and methods. We look forward to comparably exciting and original new submissions!
